# Injection of an allodermal matrix and leukocyte-rich platelet-rich plasma mixture improved the tendon integrity in cases of full-thickness common extensor tendon tears of the elbow: Two case reports

**DOI:** 10.1097/MD.0000000000041002

**Published:** 2024-12-20

**Authors:** Eun-Ji Yoon, Jung-Woo Lee, Jong-Ho Kim

**Affiliations:** a Department of Orthopedic Surgery, Yeouido St. Mary’s Hospital, College of Medicine, The Catholic University of Korea, Seoul, Republic of Korea.

**Keywords:** allodermal matrix, case report, common extensor tendon tear, injection, lateral epicondylitis, platelet-rich plasma, tennis elbow

## Abstract

**Rationale:**

Lateral epicondylitis, commonly known as tennis elbow, is a chronic condition characterized by tendinosis at the insertion site of the lateral epicondyle. Various treatment methods are available, ranging from conservative to surgical options for refractory lateral epicondylitis. Recently, platelet-rich plasma (PRP) injections have shown effectiveness for treating this condition. This study aimed to evaluate the effectiveness of injecting a mixture of allodermal matrix (ADM) and leukocyte-rich PRP (LRPRP) for tendon regeneration.

**Patient concerns:**

A 59-year-old man and a 62-year-old man, both with full-thickness tears in the common extensor tendon, presented persistent elbow pain despite undergoing several conservative treatments, including steroid injections, extracorporeal shock wave therapy, physical therapy, and medication previously.

**Diagnoses:**

The patients were diagnosed with refractory lateral epicondylitis using ultrasonography and magnetic resonance imaging (MRI).

**Interventions:**

We performed injections of a mixture of ADM and LRPRP into the full-thickness tear of the common extensor tendon.

**Outcomes:**

One year after the procedure, visual analogue scale pain scores, patient-rated tennis elbow evaluation scores, quick disabilities of the arm, shoulder, and hand scores, and Nirschl scores had all improved significantly from baseline. In case 1, MRI scans obtained at 6 and 12 months postinjection demonstrated improved tendon integrity in full-thickness tear of the common extensor tendon. In case 2, similar improvements were observed on the 6-month postinjection MRI.

**Lessons:**

Injection of a mixture of ADM and LRPRP at the site of full-thickness tears in the common extensor tendon of the elbow can enhance tendon integrity. This treatment also improves functional status in cases of recalcitrant lateral epicondylitis.

## 1. Introduction

Lateral epicondylitis, or tennis elbow, is one of the most common causes of elbow joint pain. It is a chronic condition characterized by tendinosis at the insertion site of the lateral epicondyle. It is prevalent among individuals in their 30s to 50s who overuse their forearm muscles and primarily affects the dominant arm. Although the exact cause remains unclear, it is thought that lateral epicondylitis occurs from micro-tears due to eccentric contraction of the forearm muscles under heavy loads, leading to tendon degeneration that is exacerbated by repetitive stress during the healing process.

Stepwise treatment modalities are commonly used for lateral epicondylitis; conservative treatments include medications, exercise, physical therapy, orthotic devices, and extracorporeal shock wave therapy.^[1]^ The success rate of conservative treatment is approximately 95%. If symptoms persist after 6 to 12 months of conservative treatment, surgical intervention may be considered. According to Huang et al, approximately 3% to 11% of patients ultimately undergo surgical treatment.^[2]^ Therefore, multiple institutions have conducted studies to explore more effective conservative treatments and reduce the likelihood of surgical intervention, with a focus on platelet-rich plasma (PRP) injections. PRP, which is derived from autologous blood plasma, plays a role in tendon regeneration through growth factors such as platelet-derived growth factor.^[2,3]^ Recently multiple clinical studies have reported the effectiveness of atelocollagen in tendon regeneration.^[4–6]^ Previous animal studies demonstrated significant tissue regeneration using atelocollagen^[7]^ and allodermal matrix (ADM).^[8–10]^ ADM, a human-derived material containing collagen is used in augmentation techniques during rotator cuff and Achilles tendon repairs.^[11–13]^ In this case report, we describe the results of injection with a mixture of ADM and leukocyte-rich PRP (LRPRP) for treatment of full-thickness tear of the common extensor tendon of the elbow.

## 2. Case presentation

### 2.1. Case 1

**Figure 1.**
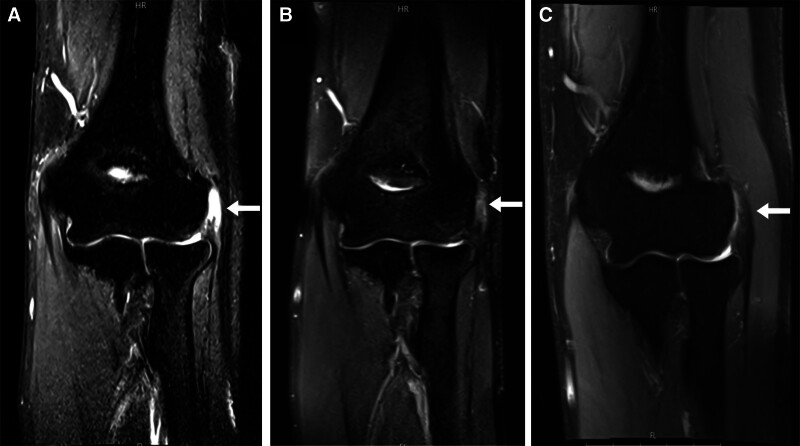
Magnetic resonance images (coronal T2-weighted fat suppressed). (A) At the initial visit, a type 3, full-thickness tear of the origin of the common extensor tendon in the left elbow (white arrow). (B) At 6 months after the first time injection, tendon integrity partially improved at the origin of the common extensor tendon (white arrow). (C) At 12 months after the first injection (at 6 months after the secondary injection), the tendon integrity of the origin of the common extensor tendon showed nearly completely improved (white arrow).

**Figure 2.**
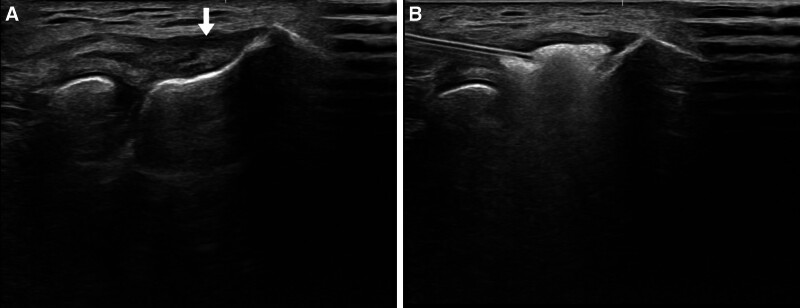
Ultrasonography images of the lateral side of the left elbow at the initial visit. (A) The attachment site of the common extensor tendon is damaged, showing heterogenous echogenicity (white arrow). (B) An ADM and LRPRP mixture injection into the damaged area of the common extensor tendon under ultrasonography. ADM = allodermal matrix, LRPRP = leukocyte-rich platelet-rich plasma.

**Figure 3.**
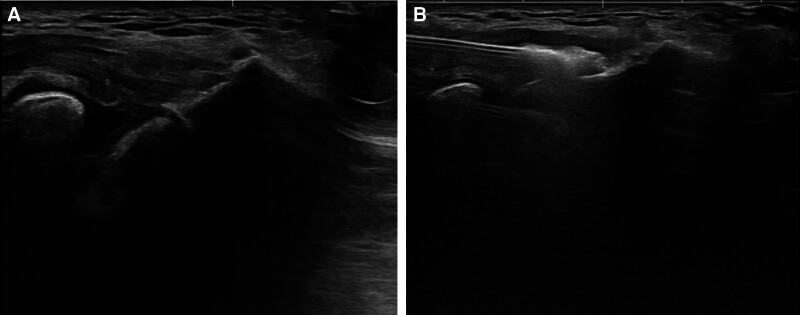
At 6 months after the first injection, ultrasonography images. (A) Reduced hypo-echogenicity at the origin of the common extensor tendon. (B) Secondary injection of an ADM and LRPRP mixture into the partially healed tendon area under ultrasonography. ADM = allodermal matrix, LRPRP = leukocyte-rich platelet-rich plasma.

A 59-year-old man visited an outpatient clinic reporting pain in his left elbow for the past year. He had received 2 corticosteroid injections and several rounds of extracorporeal shock wave therapy without significant improvement at private clinics before seeking treatment at our hospital. On initial physical examination, tenderness was noted at the lateral epicondyle. The resisted wrist extension test and Maudsley test were both positive. A type 3^[14]^ full-thickness tear of the common extensor tendon was confirmed on magnetic resonance imaging (MRI) (Fig. 1A). At that time, the patient had a visual analogue scale (VAS) pain score of 8, a patient-rated tennis elbow evaluation (PRTEE) score of 70, a quick disabilities of the arm, shoulder, and hand (Quick DASH) score of 30, and a Nirschl score of 6 (Table 1). Subsequently, we performed ultrasound-guided injection of the ADM and LRPRP mixture into the full-thickness tear of the common extensor tendon (Fig. 2). At 6 months postinjection, ultrasonography and MRI demonstrated improvement in tendon integrity, with partial tendon recovery (Fig. 1B). On physical examination, tenderness in the lateral epicondyle had decreased compared to the initial visit, and resisted wrist extension and Maudsley tests were negative. In addition, the patient’s pain and functional scores had improved significantly compared to initial scores. VAS pain score improved from 9 to 2, PRTEE score improved from 70 to 12.5, Quick DASH score improved from 30 to 8, and Nirschl score improved from 6 to 1 (Table 1). At that time, we conducted a second injection of a mixture of ADM and LRPRP into the improved tear site of the common extensor tendon under ultrasound guidance (Fig. 3). At the 12-month postprimary injection physical examination, only mild tenderness of the lateral epicondyle remained. VAS pain score was 1, PRTEE score was 6.5, Quick DASH score was 6, and Nirschl score was 1. All scores had improved from their previous visits (Table 1). There was significant improvement on the MRI performed 12 months after the first injection. The signal at the extensor carpi radialis brevis origin site showed near-complete homogeneous low intensity (Fig. 1C).

**Table 1:** Pain and functional scores of the patient in case 1.

	VAS (0–10)	PRTEE (0–100)	Quick DASH (0–36)	Nirschl (1–7)
Baseline (before the first injection)	9	70	30	6
1 month after injection	4	31.5	14	1
3 months after injection	4	21.5	9	1
6 months after injection	2	12.5	8	1
12 months after injection	1	6.5	6	1

PRTEE = patient-rated tennis elbow evaluation, Quick DASH = quick disabilities of the arm, shoulder, and hand, VAS = visual analogue scale.

### 2.2. Case 2

**Figure 4.**
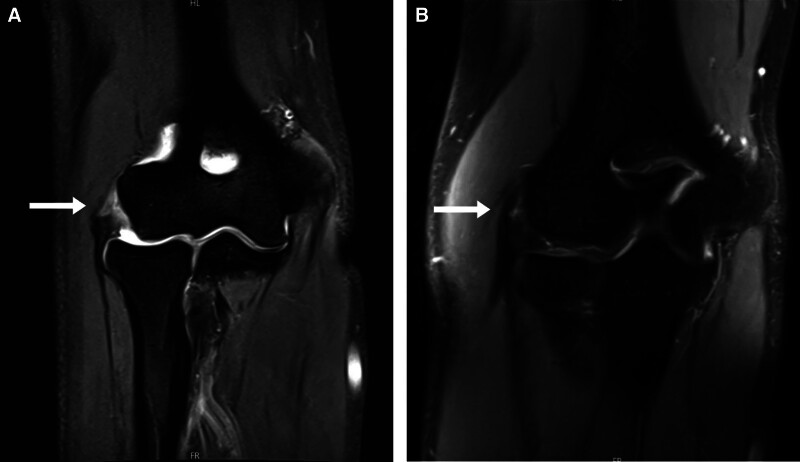
Magnetic resonance images (coronal T2-weighted fat suppressed). (A) At the initial visit, a type 3, full-thickness tear of the origin of the common extensor tendon in the right elbow (white arrow). (B) At 6 months after injection, the tendon integrity at the origin of the common extensor tendon was nearly completely improved (white arrow).

**Figure 5.**
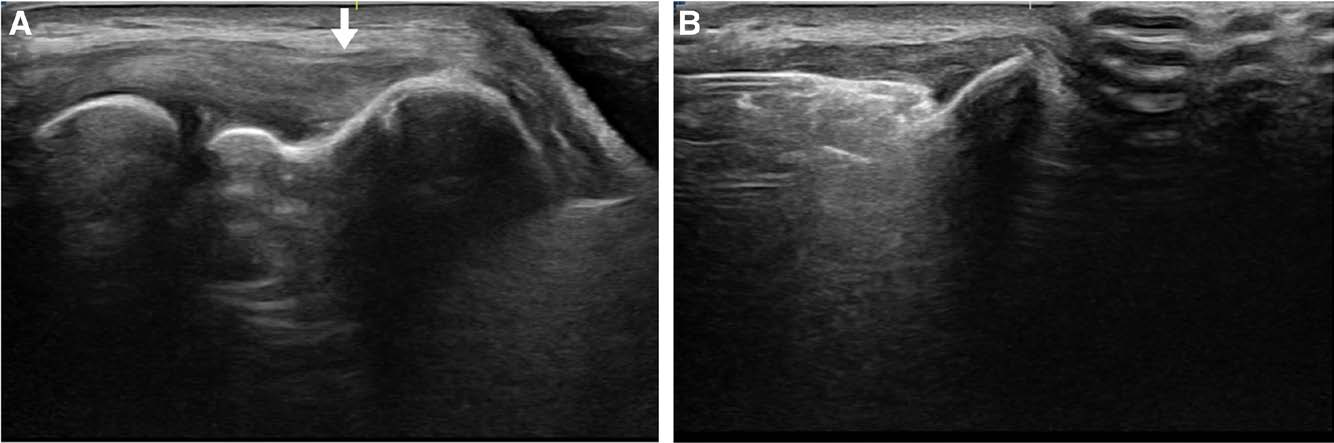
Ultrasonography images of the lateral side of the right elbow at the initial visit. (A) Focal hypo-echogenicity lesions at the common extensor tendon (white arrow). (B) An ADM and LRPRP mixture injection into the damaged area of the common extensor tendon under ultrasonography. ADM = allodermal matrix, LRPRP = leukocyte-rich platelet-rich plasma.

A 62-year-old man visited an outpatient clinic reporting pain in his right elbow for the past one and a half years. He had received 3 corticosteroid injections at a private clinic without significant improvement, prompting him to visit our hospital. MRI revealed full-thickness tear of the common extensor tendon (Fig. 4A). On initial physical examination, tenderness was noted at the lateral epicondyle, and the resisted wrist extension test and Maudsley test were positive. Consequently, we performed ultrasound-guided injection of an ADM and LRPRP mixture into the origin of the common extensor tendon (Fig. 5). At 6 months postinjection, MRI revealed near-complete tendon healing (Fig. 4B), and the patient reported a 60% subjective reduction in pain. Although the resisted wrist extension test and Maudsley test were positive, tenderness at the lateral epicondyle had resolved. Pain and functional scores were also improved from baseline. VAS pain score had improved from 7 to 3, PRTEE score from 67 to 9.5, Quick DASH score from 26 to 7, and Nirschl score from 5 to 1 (Table 2).

**Table 2:** Pain and functional scores of the patient in case 2.

	VAS (0–10)	PRTEE (0–100)	Quick DASH (0–36)	Nirschl (1–7)
Baseline (before the first injection)	7	67	26	5
1 month after injection	4	21	9	1
3 months after injection	4	20	9	1
6 months after injection	3	9.5	7	1
12 months after injection	1	6	3	1

PRTEE = patient-rated tennis elbow evaluation, Quick DASH = quick disabilities of the arm, shoulder, and hand, VAS = visual analogue scale.

## 3. Discussion

In this study, we evaluated pain relief, recovery of functional ability, and improvement in tendon integrity on MRI in patients with lateral epicondylitis treated with mixed ADM and LRPRP injection. We conducted follow-up at 1-month, 3-month, 6-month, and 12-month postinjection time periods and observed significant improvements in pain and self-reported functional ability. MRI scans obtained at 6-month and 12-month postinjection visits also showed significant improvement compared to the initial scans, suggesting tendon regeneration. Patient 1 experienced marked improvement in both pain and functional ability, with MRI indicating near-complete normalization of tendon integrity.

To the best of our knowledge, numerous institutions have studied PRP-related and collagen-related injection therapies, which are considered effective treatments for lateral epicondylitis, and found positive outcomes.^[15,16]^ However, most prior studies demonstrated pain relief and functional improvement using either PRP or collagen injection therapy alone compared with conventional steroid injection therapy. Corrado et al^[17]^ were the first to report improvements in pain and function in lateral epicondylitis after collagen injection; however, they did not actually confirm the presence of tendon regeneration on MRI. We observed improvement in both pain and function after injecting a mixture of ADM and LRPRP; in contrast to previous studies, we also objectively confirmed the evidence of tendon regeneration using MRI.

Most cases of lateral epicondylitis can be expected to respond to conservative treatment. However, 3% to 11% of patients require surgical intervention, and 9% of these patients still experience pain even after surgery.^[2]^ To address this chronic joint pain, many institutions have investigated PRP injection as a new treatment paradigm for lateral epicondylitis. This method is less prone to side effects compared to steroids and shows faster and more visible effects than other conservative treatments.^[18,19]^ PRP is autologous conditioned plasma that is immunologically versatile and contains numerous growth factors within platelet α-granules, which aid in tissue healing. Huang et al has advocated for the use of ultrasound-guided methods, highlighting its advantages such as noninvasiveness and cost-effectiveness.^[2]^ Mehdizadeh et al^[3]^ reported improved patient satisfaction with the use of ultrasound guidance compared to without it.

When tendons are injured, growth factors are released from platelet α-granules. These growth factors bind to target cells (mesenchymal stem cells, osteoblasts, fibroblasts, endothelial cells, epidermal cells), activate intracellular signaling proteins, and trigger gene expression. Furthermore, they induce cellular proliferation, matrix formation, and osteoid production or collagen synthesis, promoting tendon healing.^[3]^

In contrast to previous studies, our goal was to directly influence tendon regeneration by injecting collagen, a normal tendon component, along with LRPRP. In our 2 cases, we found that injections of an ADM and LRPRP mixture led to significant improvement in pain relief (VAS), functional and living scores (PRTEE), Quick DASH, and Nirschl scores at 1-month, 3-month, 6-month, and 12-month follow-up visits. These findings provide evidence of treatment efficacy on all subjective evaluations. Additionally, at the 6-month and 12-month follow-up visits, MRI and ultrasonography confirmed direct evidence of tendon regeneration.

Despite the potential benefits of conservative treatment, many orthopedic surgeons and their patients resort to surgical interventions (such as extensor carpi radialis brevis origin decortication) when conservative treatment fails to yield results. However, most surgical treatments are accompanied by complications unrelated to their primary purpose, placing a major burden on patients. Therefore, direct injection of collagen using ADM in addition to conventional LRPRP may represent a noteworthy new treatment approach with considerable value. This new treatment offers a potential solution to the challenges of surgical repair and provides an effective alternate intervention for lateral epicondylitis.

Our study had several limitations. First, only 2 cases were presented as the basis for the clinical effect of injectable ADM and LRPRP mixture treatment. These cases reflect some of the first of recalcitrant lateral epicondylitis at our institution. However, we expect that more positive results would be obtained with longer enrollment and follow-up periods. Second, unlike previous studies, we used an LRPRP and collagen mixture, the combined effects of which were previously unknown. Moreover, although many prior studies have used atelocollagen, we used human-derived collagen, and further large-scale studies on the individual effects of LRPRP and ADM are needed.

## 4. Conclusion

Our 2 cases suggest that injection of ADM and LRPRP mixture at the full-thickness tear site of the common extensor tendon can enhance tendon integrity and improve function in recalcitrant lateral epicondylitis.

## Author contributions

**Conceptualization:** Jong-Ho Kim.

**Data curation:** Jung-Woo Lee.

**Methodology:** Jong-Ho Kim.

**Supervision:** Jong-Ho Kim.

**Writing – original draft:** Eun-Ji Yoon.

**Writing – review & editing:** Eun-Ji Yoon, Jung-Woo Lee, Jong-Ho Kim.
